# Psychometric Properties of the General Anxiety Disorder 7-Item (GAD-7) Scale in a Heterogeneous Psychiatric Sample

**DOI:** 10.3389/fpsyg.2019.01713

**Published:** 2019-08-06

**Authors:** Sverre Urnes Johnson, Pål Gunnar Ulvenes, Tuva Øktedalen, Asle Hoffart

**Affiliations:** ^1^Modum Bad Psychiatric Center, Vikersund, Norway; ^2^Department of Psychology, University of Oslo, Oslo, Norway

**Keywords:** GAD-7, anxiety, psychometric, assessment, comorbidity

## Abstract

The GAD-7 is commonly used as a measure of general anxiety symptoms across various settings and populations. However, there has been disagreement regarding the factor structure of the GAD-7, and there is a need for larger studies investigating the psychometric properties of the measure. Patients undergoing treatment (*N* = 1201), both inpatient and outpatient patients, completed the GAD-7 at pre- and post-treatment. Measures of depression, well-being, and other anxiety measures were also completed, making it possible to investigate convergent and divergent validity. Internal consistency and convergent validity were excellent for the total sample, and there was acceptable variation related to treatment groups. We conducted an exploratory factor analysis (EFA) on a random sample (50%) of the patients at intake and then conducted a confirmatory factor analysis (CFA) to confirm the factor structure in the other part of the sample at intake. The EFA indicated a clear one-factor solution, but the one-factor solution with CFA provided a poor fit to the data. Correlating the residuals among items assessing somatic symptoms led to a good fit in a respecified CFA solution. The GAD-7 has excellent internal consistency, and the one-factor structure in a heterogeneous clinical population was supported.

## Introduction

The 7-item Generalized Anxiety Disorders Scale (GAD-7; Spitzer et al., 2006) was developed as a screener for generalized anxiety disorder (GAD) in primary care settings.

Originally, the development of the GAD-7 started with 13 items based on the criteria for GAD in the Diagnostic and Statistical Manual for Mental Disorders, Fourth Edition (DSM-IV) and other items in anxiety measures. Items were then correlated with the total score. The seven items with the highest correlation with the total 13-item scale were selected (Spitzer et al., 2006). The seven items assess (1) feeling nervous, anxious, or on edge; (2) being able to stop or control worrying; (3) worrying too much about different things; (4) trouble relaxing; (5) being restless; (6) becoming easily annoyed or irritable; and (7) feeling afraid as if something awful might happen. Even though GAD-7 was developed for GAD, it is also used in other anxiety disorders. The GAD-7 is increasingly used as a measure for anxiety in general (Beard and Björgvinsson, 2014) and in anxiety disorder research (Dear et al., 2011).

The use of GAD-7 across different anxiety disorders is warranted since GAD is an anxiety disorder with a high degree of comorbidity (Kessler et al., 2012). Further, the core feature of GAD is worry, which is a process that is found across psychological disorders and is thus transdiagnostic (Harvey et al., 2004). Psychometric evaluations of the GAD-7 suggest that it is a reliable and valid measure of GAD symptoms in the psychiatric (Kertz et al., 2013; Rutter and Brown, 2017) and general population (Löwe et al., 2008; Hinz et al., 2017) samples. The GAD-7 has demonstrated good psychometric properties, including sensitivity and specificity for diagnosing GAD (Spitzer et al., 2006). Sensitivity and specificity decrease and increase in a continuous manner, higher sensitivity is associated by a lower cut-off point, but gives lower specificity. Thus, when trying to establish a cut-off point these two factors should be balanced. In the study by Spitzer et al. (2006), 965 patients underwent diagnostic interviews to determine the presence of GAD-diagnosis. The optimal balance between sensitivity and specificity for the GAD-diagnosis was found to be a cut-off point of ≥10 (Spitzer et al., 2006). Most patients (89%) with GAD had GAD-7 scores of 10 or greater, whereas most patients (82%) without GAD had scores less than 10.

The psychometric properties of the GAD-7 have also been examined in a heterogeneous sample of different diagnoses. Beard and Björgvinsson (2014) found good internal consistency and convergent validity but poor specificity and a high false positive rate for specific anxiety disorders. Also, the proposed cutoff by Spitzer et al. (2006) of ≥10 was only partly supported with a sensitivity of 74% and specificity of 54%. Kroenke et al. (2007) found that the GAD-7 performed well as a screener for GAD, post-traumatic stress disorder (PTSD), social anxiety disorder (SAD), and panic disorder (PD) in primary care patients and proposed a score of 8 as a cutoff score with a positive likelihood ratio above 3.

Also, the factor-structure of GAD-7 has been investigated. Löwe et al. (2008), *N* = 5030, reported a one-dimensional factor structure in a national representative study. Kertz et al. (2013), *N* = 232, used confirmatory factor analysis (CFA) in an acute psychiatric sample in an attempt to replicate the unidimensional factor found by Löwe et al. (2008), but were not able to support the structure due to three items measuring bodily symptoms (items 4–6). Beard and Björgvinsson (2014), *N* = 1082, investigated the GAD-7 in a heterogeneous psychiatric sample. They proposed a two-factor structure for the GAD-7 using exploratory factor analysis (EFA), one factor reflecting bodily symptoms (items 4–6) and the other factor assessing the cognitive and emotional experience of anxiety (items 1–3 and 7). However, a recently published study used CFA and found evidence for a unidimensional factor, after accounting for the additional covariance found between items, 4, 5, and 6 (Rutter and Brown, 2017). This study was based on 536 outpatients presenting at a specialty clinic for anxiety and mood disorders. However, sensitivity and specificity could not be balanced at any cutoff point. In summary, the GAD-7 is a well-established measure that has proven to be highly reliable. However, further evaluation of the measurement is needed to establish the orderly factor-structure and cutoff.

This paper aimed to investigate the internal consistency, reliability, and validity in a large sample of heterogeneous psychiatric patients. If GAD-7 is going to be used as a measure of anxiety in general, further psychometric evaluations are needed. Furthermore, we wanted to extend previous research by also investigating how the GAD-7 operates in a population with chronic psychological disorders (inpatient) and a general outpatient population. We also wanted to study the appropriate cutoff for the GAD-7 total score for anxiety disorders, balancing sensitivity and specificity, since there are inconsistent findings in the literature. The Beck Anxiety Inventory was used as a criterion variable, because it measures anxiety symptoms in general, and could therefore be viewed as a transdiagnostic measure for anxiety. Finally, we wanted to conduct an exploratory and CFA of the GAD-7 items. Based on previous investigations, we expected the GAD-7 to have excellent reliability and convergent and discriminant validity and to have a unidimensional structure. To test for discriminant validity, we predicted that the scores of the patients with anxiety disorders would be higher compared to a more general psychiatric population.

## Materials and Methods

### Participants and Procedure

There were 1201 respondents at the start of therapy, and 883 at the termination. Of those reporting gender, 797 (72%) were female, and 303 (28%) were male. A total of 662 (55.1%) patients provided some information about demographics, mainly patients from the inpatient units. The average duration of psychological symptoms for the patients was 15.8 years (*SD* = 11.4, *N* = 531). Mean age was 40.9 years (*SD* = 11.8, *N =* 627). Exclusion criteria for all units include active substance abuse, active suicidal ideation, or active psychosis.

The patients were admitted to one of five inpatient or four outpatient units. The inpatient units were located at the same psychiatric hospital, which contains highly specialized treatment for eating disorders, anxiety disorders, depressive disorders, trauma disorders, as well as family therapy. The eating disorder unit offers either cognitive behavioral therapy (CBT) or compassion-focused therapy, each lasting 12 weeks. The anxiety disorder unit offers either CBT or metacognitive therapy (MCT), both treatments lasting 8 weeks. The depression unit offers 12-week short-term dynamic psychotherapy. The trauma unit uses integrative trauma-focused therapy with treatment lasting 12 weeks. The family therapy unit uses systemic approaches with treatment lasting 12 weeks. The outpatient units consist of a trauma therapy unit providing stabilizing and a psycho-educative group therapy lasting from 12 to 20 weeks, a community-based outpatient unit and two low-threshold outpatient units aimed at early intervention and prevention, all three with treatment length depending on the presenting problem. The patients at the low-threshold units were not asked to provide demographic data, and no journal records were kept, in accordance with the low-threshold program. The therapists (psychologists, psychiatrists, nurses, and social workers) at the different units received regular supervision. For the inpatient units and the trauma and community-based outpatient clinic, the data were collected using an online survey system.

For the low-threshold units, the data were collected using paper and pencil and the instruments. The data were collected as part of a standardized assessment package that all patients referred to the treatment units were asked to fill out. The participants provided written informed consent for the use of their data from the standardized assessment package in future research. Anonymized data were used in the evaluation of the GAD-7. The data collection procedure was discussed and approved by the Norwegian regional ethical committee (REK south-east) and an internal review board at Modum Bad Hospital. Another written consent was not required for this particular study since the data used were part of a standardized assessment package, and they were anonymized. The patients were asked to fill out the questionnaires at the start and termination of therapy. A new Norwegian translation of GAD-7 was used. The instruments were translated from English to Norwegian by the author SJ (psychologist, Ph.D.), and then back-translated by an independent native English-speaker with an MD and who had practiced as a psychiatrist in Norway and spoke Norwegian fluently. The resulting back translation had only minor differences from the original version, and the nuances were resolved by discussing different wording and phrases with native English and Norwegian speakers (PU, TØ and AH).^1^

### Measures

#### Generalized Anxiety Disorder 7

GAD-7 (Spitzer et al., 2006) consists of seven items measuring worry and anxiety symptoms. Each item is scored on a four-point Likert scale (0–3) with total scores ranging from 0 to 21 with higher scores reflecting greater anxiety severity. Scores above 10 are considered to be in the clinical range (Spitzer et al., 2006). The GAD-7 has shown good reliability and construct validity (Kroenke et al., 2007; Löwe et al., 2008).

#### Patient Health Questionnaire 9

PHQ-9 (Kroenke et al., 2001) consists of nine items measuring depressive symptoms corresponding to the diagnostic criteria for major depressive disorder. Each item is scored on a four-point Likert scale (0–3) with scores ranging from 0 to 27, with higher scores reflecting greater depression severity. Scores above 10 are considered to be in the depressive area. PHQ-9 has shown good psychometric properties (Kroenke et al., 2001).

#### Symptom Check List 90-R

SCL-90 (Derogatis, 1983) is a broad measure of symptom distress relevant for psychotherapy. Each item is scored on a five-point Likert scale (0–4). SCL-90 produces nine subscales, including a subscale for anxiety and depression, along with a Global Severity Index (GSI; Derogatis, 1983; Derogatis and Savitz, 2000). SCL-90 has been found to be having good psychometric properties (Schmitz et al., 2000).

#### The Short Form 36 Health Survey (SF-36; Ware et al., 1993)

SF-36 measures patients’ health status. One item assesses perceived change in health status, and the remaining form eight subscales and two summary scales^2^. The items use Likert-type scales, some with five or six points and others with two or three points. Reliability and validity have been demonstrated extensively in multiple populations (Ware, 1999). The instrument has known utility functions and is, therefore, often used in cost-utility analysis (Ware et al., 1993). In the calculation of SF-36, norm data from 2009 were used.

#### Beck Depression Inventory II (BDI-II; Beck et al., 1996)

BDI-II consists of 21 items and measures depressive symptoms. BDI uses a four-point Likert scale (0–3) with scores ranging from 0 to 63. Scores above 16 are considered clinical cutoff based on the norms by Beck et al. (1996). The scale has demonstrated sufficient reliability and validity (Osman et al., 2004; Lykke et al., 2008).

#### Beck Anxiety Inventory (BAI; Beck et al., 1988)

BAI consists of 21 items and measures anxiety symptoms. It uses a four-point Likert scale (0–3) with scores ranging from 0 to 63. Scores above 15 are considered a clinical cutoff (Gillis et al., 1995). The scale has been found to have good reliability and validity (Osman et al., 1997).

### Data Analysis

Cronbach’s alpha was used to investigate internal consistency at intake and post-treatment. Second, Pearson correlations were calculated to investigate convergent validity. Discriminant validity was conducted trough independent *t*-tests. Receiver operating characteristics analysis (ROC analysis) was conducted to check for specificity and sensitivity using the BAI as the cutoff on those patients who had completed the BAI (*n* = 229). The cutoff score of 15 was calculated based on the norms from Gillis et al. (1995). Also, we assessed the sensitivity by conducting a paired-sample *t*-test of GAD-7 score before and after treatment. The described analysis was conducted with SPSS 25.

We decided to split the factor analysis in two by designating a random half as exploratory and the other half as confirmatory. This allows for a within-study replication of structural or other key analyses. We conducted an EFA on a random sample (50%) of the patients at intake and then conducted a CFA to confirm the factor structure in the other part of the sample at intake. A two-factor solution was also tested. The analyses were conducted with Mplus version 8. Maximum likelihood estimation was used. The present modeling approach was undertaken in two phases. First, a confirmatory one-factor model was fitted to the data. The second phase involved the use of multiple indicators, multiple causes (MIMIC) modeling to investigate whether the latent factor mediates the effect of the observed severity group on the latent construct of the GAD-7 in a heterogeneous sample of Norwegian patients at the start of treatment. The severity was coded 1 = high severity, including inpatient treatment of eating disorders, anxiety disorders, depressive disorders, and trauma disorders, and 2 = low severity, including inpatient family treatment and the outpatient units. A direct path to the latent construct indicates the effects of the group contrast. There are several criteria to evaluate models. Multiple indices were used because they provide different information (i.e., absolute fit, fit adjusting for model parsimony, fit relative to a null model), and more indices give a more conservative and reliable evaluation of the model fit (Brown, 2015). The chi-square distribution for goodness of fit evaluates the difference between the observed data and model prediction. For the comparative fit index (CFI; Hu and Bentler, 1999) and the Tucker-Lewis Index (TLI; Schumacker and Lomax, 2004), a value of 0.95 suggests acceptable fit. For root mean square error of approximation (RMSEA; Browne and Cudeck, 1993), values in the range of 0.00 to 0.05 indicate close fit, those between 0.05 and 0.08 indicate fair fit, and those between 0.08 and 0.10 indicate mediocre fit. RMSEA values above 0.10 indicate poor fit. Standardized root mean square residual (SRMR) is an absolute measure of fit and is defined as the standardized difference between the observed correlation and the predicted correlation. A value below 0.08 is generally considered a good fit (Hu and Bentler, 1999). Bayesian information criterion (BIC; Schwarz, 1978) and Akaike information criterion (AIC; Akaike, 1974) were used to compare the one and two-factor solution.

## Results

### Reliability

Cronbach’s alpha was calculated for all scales except SF-36. The alpha was also tested with each of the items removed from the analysis to see if the removal of one item increased the alpha. No alpha increased as a result of this procedure. The alpha for GAD was α = 0.88 at the start of therapy for the total sample and ranged from 0.83 to α = 0.93 across units and pre- and post-treatment. The alpha for BAI was α = 0.93 at the start of therapy for the total sample, and ranged from α = 0.81 to α = 0.99 across units and pre- and post-treatment (see Table 1 for details on alpha for all instruments at pre- and post-treatment).

**TABLE 1:** Cronbach’s alpha at pre- and post-treatment across different treatment sites.

**Treatment sites**		**GAD**	**BAI**	**BDI**	**PHQ**	**GSI**	**SCL DEP**	**SCL ANX**
Total (*N* = 2084)	Pre	0.877	0.934	0.909	0.875	0.974	0.889	0.890
	Post	0.913	0.951	0.941	0.907	0.983	0.932	0.919
Community-based outpatient clinic (*N* = 390)	Pre	0.866		0.918	0.889	0.973	0.904	0.863
	Post	0.891		0.938	0.887	0.977	0.932	0.874
Trauma outpatient clinic (*N* = 206)	Pre	0.860		0.909	0.846	0.973	0.846	0.893
	Post	0.903		0.926	0.889	0.977	0.886	0.925
Inpatient trauma clinic (*N* = 275)	Pre	0.866		0.892	0.849	0.964	0.860	0.871
	Post	0.887		0.934	0.887	0.978	0.912	0.896
Inpatient depression clinic (*N* = 286)	Pre	0.858		0.882	0.858	0.963	0.855	0.873
	Post	0.897		0.933	0.866	0.975	0.912	0.898
Inpatient anxiety clinic (*N* = 280)	Pre	0.871	0.916	0.910	0.855	0.970	0.877	0.851
	Post	0.932	0.946	0.946	0.913	0.985	0.944	0.917
Inpatient eating disorder clinic (*N* = 239)	Pre	0.825		0.899	0.860	0.973	0.887	0.887
	Post	0.894		0.933	0.899	0.977	0.924	0.893
Inpatient family unit (*N* = 206)	Pre	0.906		0.936	0.898	0.980	0.914	0.909
	Post	0.913		0.947	0.897	0.985	0.947	0.912
Low-threshold clinic 1 MB (*N* = 138)	Pre	0.902	0.945	0.906	0.849			
	Post	0.916	0.946	0.981	0.973			
Low-threshold clinic 2 NOT (*N* = 64)	Pre	0.876	0.927	0.894	0.884			
	Post	0.891	0.814	0.935	0.866			

*Low-threshold clinic 1 MB, Low-threshold clinic 1 Modum Bad; Low-threshold clinic 2 NOT, Low-threshold clinic 2 Notodden; GAD, Generalized Anxiety Disorder Scale – 7; BAI, Beck Anxiety Inventory; BDI, Beck Depression Inventory; PHQ, Patient Health Questionnaire – 9; GSI, Global Severity Index (SCL-90); SCL DEP, Symptom Checklist subscale depression; SCL ANX, Symptom Checklist subscale anxiety; Total number of participants pre- and post- are given in parenthesis.*

### Validity

The correlations between the instruments were calculated at both time points. As expected, the GAD had a high positive correlation with BAI, *r* = 0.69, *p* < 0.01, SCL-90 anxiety, *r* = 0.76, *p* < 0.01, and SCL-90 GSI, *r* = 0.72, *p* < 0.01, correlated positively but with a lower or similar coefficient with PHQ, *r* = 0.69, *p* < 0.01, SCL-90 depression, *r* = 0.64, *p* < 0.01, and BDI-II, *r* = 0.58, *p* < 0.01, and correlated negatively with the SF-36 mental component, *r* = −0.59, *p* < 0.01, and SF-36 physical component, *r* = −0.17, *p* < 0.01 at time point 1 for the total sample (see Tables 2, 3 for all correlations at both time points across the units). To test for discriminant validity, we compared the GAD-7 scores of a severe population with a population that has less distress. Thus, an independent *t*-test was performed comparing the GAD-7 scores for patients at the anxiety unit with patients at the family unit at intake to test the discriminant validity of GAD-7. As predicted, patients at the anxiety unit scored significantly higher on the GAD-7 (*M* = 11.78; *SD* = 5.07; *N* = 131) compared to the family unit (*M* = 6.81; *SD* = 4.87; *N* = 111), *t*(240) = 7.75, *p* ≤ 0.01, two-tailed. The difference in effect was large using Hedges’ *g* = 1.00. These findings indicate that GAD-7 discriminates well between an anxious and a more general psychiatric population.

**TABLE 2:** Correlation between measures at pre-treatment.

	**BDI**	**BAI**	**PHQ**	**GSI**	**SCL depression**	**SCL anxiety**	**GAD**	**SF-36 pcs**	**SF-36 mhs**
BDI	1								
BAI	0.551^∗∗^	1							
PHQ	0.775^∗∗^	0.540^∗∗^	1						
GSI	0.767^∗∗^	0.711^∗∗^	0.757^∗∗^	1					
SCL depression	0.802^∗∗^	0.610^∗∗^	0.780^∗∗^	0.874^∗∗^	1				
SCL anxiety	0.605^∗∗^	0.757^∗∗^	0.634^∗∗^	0.875^∗∗^	0.720^∗∗^	1			
GAD	0.576^∗∗^	0.685^∗∗^	0.685^∗∗^	0.722^∗∗^	0.636^∗∗^	0.756^∗∗^	1		
SF-36 pcs	−0.220^∗∗^	−0.278^∗∗^	−0.249^∗∗^	−0.350^∗∗^	−0.240^∗∗^	−0.316^∗∗^	−0.165^∗∗^	1	
SF-36 mhs	−0.697^∗∗^	−0.444^∗∗^	−0.684^∗∗^	−0.633^∗∗^	−0.693^∗∗^	−0.552^∗∗^	−0.585^∗∗^	−0.049	1

*^∗∗^*p* < 0.01. BDI, Beck Depression Inventory; BAI, Beck Anxiety Inventory; PHQ, Patient Health Questionnaire – 9, GSI, Global Severity Index (SCL-90); SCL DEP, Symptom Checklist subscale depression; SCL ANX, Symptom Checklist subscale anxiety; GAD, Generalized Anxiety Disorder Scale – 7; SF-36 pcs, short form health survey 36 physical sub-scale; SF-36 mhs, short form health survey 36 mental sub-scale.*

**TABLE 3:** Correlation between measures at post-treatment.

**Measures**	**BDI**	**BAI**	**PHQ**	**GSI**	**SCL depression**	**SCL anxiety**	**GAD**	**SF-36 pcs**	**SF-36 mhs**
BDI	1	0.799^∗∗^	0.859^∗∗^	0.831^∗∗^	0.856^∗∗^	0.723^∗∗^	0.750^∗∗^	−0.356^∗∗^	−0.769^∗∗^
BAI		1	0.797^∗∗^	0.899^∗∗^	0.713^∗∗^	0.823^∗∗^	0.835^∗∗^	−0.303^*^	−0.637^∗∗^
PHQ			1	0.848^∗∗^	0.857^∗∗^	0.760^∗∗^	0.782^∗∗^	−0.308^∗∗^	−0.834^∗∗^
GSI				1	0.902^∗∗^	0.915^∗∗^	0.820^∗∗^	−0.453^∗∗^	−0.696^∗∗^
SCL depression					1	0.823^∗∗^	0.771^∗∗^	−0.325^∗∗^	−0.780^∗∗^
SCL anxiety						1	0.816^∗∗^	−0.387^∗∗^	−0.659^∗∗^
GAD							1	−0.278^∗∗^	−0.729^∗∗^
SF-36 pcs								1	0.117
SF-36 mhs									1

*^∗∗^*p* < 0.01. BDI, Beck Depression Inventory; BAI, Beck Anxiety Inventory; PHQ, Patient Health Questionnaire – 9; GSI, Global Severity Index (SCL-90); SCL DEP, Symptom Checklist subscale depression; SCL ANX, Symptom Checklist subscale anxiety; GAD, Generalized Anxiety Disorder Scale – 7; SF-36 pcs, Short form health survey 36 physical sub-scale; SF-36 mhs, Short form health survey 36 mental sub-scale.*

The ROC curve graphically displays the trade-off between sensitivity and specificity and is useful in assigning the best cutoffs for clinical use. The analysis indicated gave a sensitivity of 0.92 and a specificity of 0.7 for a cutoff of 8. Of those patients who scored >8 on the GAD-7 scale, 83.3% had an anxiety problem as classified by the BAI with a cutoff point of 15. The area under the ROC curve was 86.9%. Thus, the ROC analysis showed that the best cutoff point was a score of 8 on the GAD-7.

The average GAD-7 score at pre-treatment was 10.3 (5.2) and at post-treatment 7.7 (5.3). The difference between the two scores was significant using a paired sample *t*-test, *p* ≤ 0.01, Cohen’s *d* = 0.5, which indicates a medium effect size from pre to post-treatment (Cohen, 1988). Thus, the GAD-7 was sensitive to change.

### Factor Structure

The EFA was conducted on a random sample of the patient’s intake scores of the GAD-7 (50% of the scores) to investigate the factor structure that should be further investigated in the CFA in the second part of the sample at intake. According to a scree plot, Kaiser criterion (eigenvalues above 1) and horn parallel analysis (Horn, 1965), a one-factor structure best fits the data. Using EFA with varimax rotation (*N* = 576), the factor loadings on the seven items varied from 0.44 to 0.87. One factor had an initial eigenvalue of 4.2 and two factors of 0.8. The 95th percentile estimated eigenvalues from the parallel analysis indicated that a two-factor solution should have an eigenvalue above 1.1.

**FIGURE 1:**
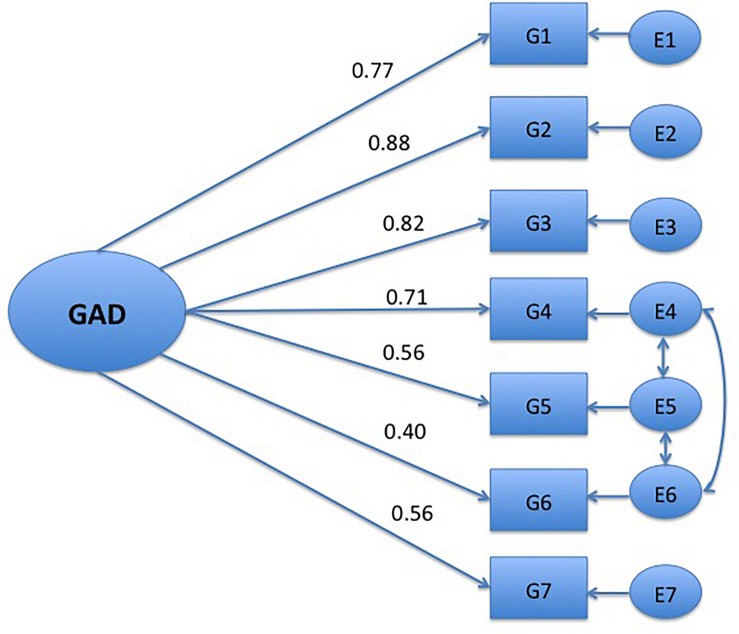
One-factor model of the GAD-7. Only significant paths, *p* = 0.05 are shown. G1, nervous; G2, control of worry; G3, worry; G4, trouble relaxing; G5, restless; G6, irritable; G7, afraid.

Confirmatory factor analysis was used to investigate further and confirm the one-factor structure of the GAD-7. The one-factor model provided a poor fit for the other split-half sample [χ^2^_14_(*N* = 562). 125.865; *p* = 0.00; CFI = 0.94; TLI = 0.91; RMSEA = 0.119; (0.101–0.139); SRMR = 0.039]. Thus, the one-factor solution was not confirmed. However, when evaluating the modification indices, evidence of correlated residuals for items 4, 5, and 6 where found, similar to the Rutter and Brown (2017) study. The correlated residuals could be expected since all the items measure bodily symptoms. Thus, the CFA was specified again by freely estimating the error covariances of these items (see Figure 1). The revised model gave a better model fit, [χ^2^_11_(*N* = 562). 43.115; *p* = 0.00; CFI = 0.98; TLI = 0.97; RMSEA = 0.07; (0.05–0.01); SRMR = 0.019, AIC = 9227.385, BIC = 9331.341]. We also tested a two-factor solution of the GAD-7, separating item 4–6 as a separate factor. However, the model fit were worse then the revised one-factor solution, [χ^2^_15_(*N* = 562). 91.452; *p* = 0.00; CFI = 0.96; TLI = 0.94; RMSEA = 0.10; (0.08–0.011); SRMR = 0.14, AIC = 9267.722, BIC = 9354.352]. Thus in the subsequent MIMIC analysis, the revised one-factor solution was used.

The response indicators defining the one-factor model reported in Figure 1 were inserted in the entire MIMIC model. The contrast variable represented severity and formed the set of a potential subgroup effect. The contrast variable was regressed on the latent construct (GAD). The MIMIC model indicated a better fit than the CFA-model: [χ^2^_17_(*N* = 562). 56.593; *p* = 0.00; CFI = 0.98; TLI = 0.96; RMSEA = 0.06; (0.05–0.080; SRMR = 0.025]. Thus, including a covariate in the measurement model, which indicates treatment severity, improved the model fit. The negative estimate between the covariate and the latent construct (−0.07) indicate that the patients from treatment sites with low severity had lower scores than patients from inpatient units with high severity. However, the estimate was not significant, *p* = 0.46.

## Discussion

The GAD-7 demonstrated good internal consistency and convergent validity as hypothesized. The alphas were all above 0.82 at intake and post-treatment, and correlations were large with other measures of anxiety and well-being, indicating high reliability and validity. The correlations found in this study, ranging from *r* = 0.70 for anxiety disorders and from *r* = 0.62 for related construct are comparable to other studies (Spitzer et al., 2006; Löwe et al., 2008). Thus, the GAD-7 demonstrated convergent validity. Furthermore, GAD-7 discriminates well between an anxious and a more general psychiatric population indicating discriminant validity.

The ROC analyses identified 8 as the optimal point for specificity and sensitivity. A cutoff point of 8 represents a threshold for identifying possible cases in which further questioning to determine the presence and type of anxiety disorder may be warranted. This is in accordance with the results from Kroenke et al. (2007) who also found a cutoff score of 8. Furthermore, using a cutoff score for further evaluation and not for diagnostic purposes is in accordance with a recent study recommending that the GAD-7 should not be used to screen for GAD when there are clinical samples characterized by high general distress (Rutter and Brown, 2017). However, the cutoff found was used with BAI as a criterion variable. Even though the BAI has been found to specify between anxiety disorders, it has been argued that the measure does not capture the whole range of anxiety disorders due to the emphasis on somatic symptoms (Creamer et al., 1995). Thus, the proposed cutoff point must be interpreted with caution and should be replicated with formal diagnostics.

The one-factor solution found in the EFA could not be confirmed with the CFA. The factor loading for item 6 (irritability) was especially low. However, a revised model encompassing the residuals on the somatic items 4–6 improved the fit. Previous research has identified a two-factor model of GAD-7 (Beard and Björgvinsson, 2014), but this was based on EFA, and as discussed by Rutter and Brown (2017), what would be represented as a method effect in CFA could emerge as additional but trivial factors in EFA. To summarize, these results replicate the results from Kertz et al. (2013), Rutter and Brown (2017) and supports the unidimensional construct of the GAD-7 in a psychiatric sample with a high degree of heterogeneity.

There was no indication of a group effect, indicating that the GAD-7 did not vary across treatment sites. The model fit was slightly improved when this covariate was entered in a MIMIC model. However, the negative estimate indicated that patients with high severity had higher scores. The inpatient sample used in this study has a long duration of psychological disorders and chronicity, as previously documented in previous studies (Johnson et al., 2017). The chronicity of psychological disorder may thus lead to lower scores on the GAD-7 compared to more acute distress measured in the outpatient sample.

The clinical implication of this study is that the GAD-7 can be used as an index of anxiety severity. The scores eight and above could indicate that an anxiety disorder is present and warrants further investigation. Also, the measures can be used in diverse samples, which is an advantage, since patients in clinical practice are often complex and comorbid. The GAD-7 has several advantages: it is easy to use, has clear psychometric properties, and consists of only seven items. Thus, it can be safely adopted by clinicians.

Strengths of the current study include that a large, heterogeneous psychiatric sample was studied and several measures were used to investigate psychometric properties of the GAD-7. Furthermore, this study used a large sample to validate and confirm the EFA. However, several limitations should also be addressed. First, we did not have specific diagnostic information for much of the sample. Since the BAI was used to calculate the cutoff for an anxiety disorder, we could not investigate whether the GAD-7 performed differently with different specific diagnostic groups. Second, there was a lack of demographic information available, making it difficult to investigate whether the GAD-7 performs differently based on demographic variables. However, information regarding the treatment site made it possible to separate those treated at in-patient and out-patient units and discern whether the patients were high or low on clinical severity. Third, differences from published findings using other versions of this measure may be due to language or cultural differences.

This study supports the GAD-7 as an efficient and valid self-report anxiety measure, and that the measure could be used to evaluate anxiety symptoms in heterogeneous samples.

## Ethics Statement

All procedures performed in studies involving human participants were in accordance with the ethical standards of the institutional and/or national research committee and with the 1964 Helsinki declaration and its later amendments or comparable ethical standards. Anonymised data were used in the evaluation of the GAD-7. The data collection procedure was discussed and approved by the regional ethical committee in Norway and internal review board at Modum Bad hospital. For this type of study formal consent is not required.

## Author Contributions

SJ was responsible for the data analysis, interpretation, drafting, and revising the work. SJ, PU, and AH translated the questionnaires. SJ and PU collected the data. TØ participated in the interpretation and revision process of the manuscript. All authors gave their final approval of the version to be published.

## Conflict of Interest Statement

The authors declare that the research was conducted in the absence of any commercial or financial relationships that could be construed as a potential conflict of interest.

## Footnotes

1 An alternative translation of the instruments has been published before the translation presented here, but the alternative translation was done without including mental health professionals and deviates from the translation used in this manuscript in important areas. The owner of the instrument has been informed about this but has at the time of the preparation of this manuscript not corrected the translation.

2 SF-36 is copyrighted, and analysis has to be done by the copyright owner. The copyright owner was not able to provide us with the item scores for the subscales or numbers for internal consistency in our sample. The reader is referred to Sf36.org and optum.com for the copyright owners own information or Thombs, Hudson, Schieir, Taillefer and Baron (2008) regarding the reliability of the scale.
